# Long non-coding RNA ADAMTS9-AS1 attenuates ferroptosis by Targeting microRNA-587/solute carrier family 7 member 11 axis in epithelial ovarian cancer

**DOI:** 10.1080/21655979.2022.2049470

**Published:** 2022-03-21

**Authors:** Li Cai, Xiaoqing Hu, Lu Ye, Pingjuan Bai, Youkun Jie, Kuanyong Shu

**Affiliations:** a Department of gynecologic oncology, Maternal and Child Health Affiliated Hospital of Nanchang University Nanchang 330006, Jiangxi, China; b Department of Pathology, Maternal and Child Health Affiliated Hospital of Nanchang University Nanchang 330006, Jiangxi, China

**Keywords:** Long non-coding RNA ADAMTS9-AS1, micoRNA-587, solute carrier family 7 member 11, epithelial ovarian cancer, ferroptosis

## Abstract

Epithelial ovarian cancer (EOC) accounts for approximately 90% of all ovarian cancer cases and is the most common cause of gynecological cancer death. Understanding the molecular mechanisms of EOC will help develop better diagnostics and more effective treatments. This study aimed to investigate whether long non-coding RNA ADAMTS9-AS1 (ADAMTS9-AS1) could regulate solute carrier family 7 member 11 (SLC7A11) expression and inhibit ferroptosis by sponging micoRNA-587 in EOC progression. Quantitative real-time polymerase chain reaction (qRT-PCR) and western blotting results showed that ADAMTS9-AS1 expression was elevated in EOC cells; microRNA-587 expression was up-regulated and SLC7A11 expression was down-regulated after knocking down ADAMTS9-AS1 by transfection with siRNAs; however, microRNA-587 inhibitor reversed SLC7A11 expression in ADAMTS9-AS1 knocking down cells. Ferroptosis related marker detection and cell function assay confirmed that knocking down ADAMTS9-AS1 inhibited EOC cells proliferation and migration by promoting ferroptosis. Overexpression of micoRNA-587 also promoted ferroptosis while inhibited cells proliferation and migration in EOC cells. Additionally, micoRNA-587 inhibitor reversed the effect of ADAMTS9-AS1 silence on the ferroptosis and cell function. Moreover, dual-luciferase reporter gene assay and RNA immunoprecipitation assay confirmed that miR-587 was as a sponge for ADAMTS9-AS1 and SLC7A11. In conclusion, our study found that ADAMTS9-AS1 attenuated ferroptosis by targeting miR-587/SLC7A11 axis in EOC. Our study provides a new therapeutic target for EOC.

## Graphical abstract

**Figure:**
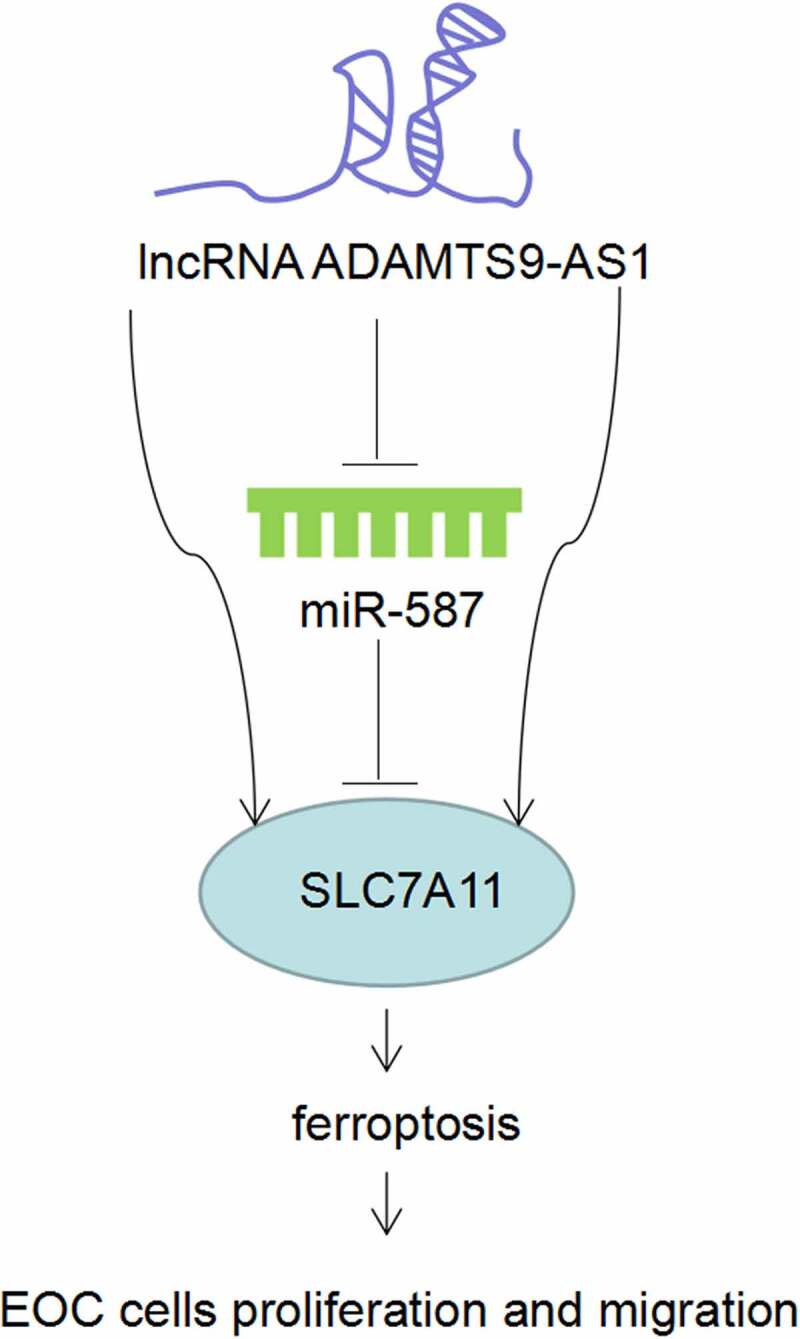

## Introduction

Ovarian cancer (OC) is a silent cancer, and the deadliest gynecological malignant tumor [1]. Epithelial ovarian cancer (EOC) accounts for about 90% of all OC cases, usually appearing in advanced stages, and is the most common cause of death from gynecological cancer [2]. In which, high grade serous ovarian cancer is the most lethal gynecologic cancer [3,4]. Despite advances in surgical techniques and conventional chemotherapy, EOC patients often relapse due to chemotherapy resistance within a few years of initial treatment, and the long-term survival rate of EOC patients (more than 5 years) is less than 30% [5,6]. EOC pathogenesis is a complex biological process involving gene and epigenetic changes [7,8]. Therefore, more extensive studies are urgently needed to better understand the molecular mechanisms underlying EOC progression.

Long non-coding RNAs (lncRNAs) are a class of non-coding RNA molecules with transcripts longer than 200 nt [9]. More and more evidences indicate that lncRNAs play a vital regulatory role in EOC [^10–12^]. Mechanistically, lncRNAs can directly adsorb microRNAs (miRNAs), thus regulating gene expression through weakening miRNA-mediated gene expression inhibition [12,13]. Therefore, exploring tumor-related lncRNAs and miRNAs, and competing endogenous RNAs (ceRNAs) theory, may be a feasible approach to understand EOC progression mechanism. LncRNA ADAMTS9-AS1 (ADAMTS9-AS1) could play an essential role as a cancer-promoting or cancer-suppressing molecule in various cancers, such as in colorectal cancer, ADAMTS9-AS1 promoted cell proliferation and epithelial-mesenchymal transition [14]. Additionally, previous study has found that ADAMTS9-AS1 expression was elevated in EOC [10]. However, the specific mechanism that plays a role in EOC has not been elucidated. Before conducting experiment, we used miRanda-3.3a software predicted that ADAMTS9-AS1 had binding site with miR-587. miR-587 has been reported to promote or inhibit cancer in different tumors, including prostate cancer [15], non-small-cell lung cancer [16], hepatocellular carcinoma [17], etc. However, its function in EOC has not been reported.

Before conducting experiment, we also used miRanda-3.3a predicted that downstream targets of miR-587 was SLC7A11. SLC7A11, also called xCT, was the glutamate/cystine antiporter solute carrier family 7 member 11 [18]. Exploring SLC7A11 regulation mechanism has always been a significant research focus, and several studies on SLC7A11 have been involved. SLC7A11 has been reported to be overexpressed in cancer and associated with poor prognosis in patients. These diseases included melanoma [19], glioma [20], non-small cell lung cancer [21], etc. In OC, SLC7A11 was reported to be an independent risk prognostic factor for overall survival [22]. Moreover, SLC7A11, as a key suppressor of ferroptosis, has been reported to play a role in inhibiting ferroptosis in glioma and OC [23,24]. But it is unknown whether ADAMTS9-AS1 plays a role in EOC progression by regulating miR-587/SLC7A11 axis and thereby inhibiting ferroptosis.

Based on the above background, we hypothesis that ADAMTS9-AS1 could regulate miR-587/SLC7A11 axis and inhibit ferroptosis in EOC progression. To verify this hypothesis, we firstly investigate the function of ADAMTS9-AS1, and then confirmed the relationship between ADAMTS9-AS1, miR-587, and SLC7A11. Finally, rescue experiments were performed to verify the potential regulation mechanism. Our study was intended to provide new drug targets for EOC diagnosis and treatment.

## Materials and methods

### Cell culture

The human ovarian surface epithelial cell line OSE (#7310, ScienCell Research Laboratories) were grown in ovarian epithelial cell medium (ScienCell Research Laboratories). EOC cell lines are high grade serous ovarian cancer, including ES-2, OVCAR3, and CAOV-3. ES-2 (#CRL-1978, ATCC) were cultured in McCoy<apos;>s 5A Medium that was supplemented with 10% fetal bovine serum (FBS) (Gibco, Thermo). OVCAR3 (#HTB-161, ATCC) were maintained in 20% FBS-supplemented RPMI-1640 medium (Gibco, Thermo) and bovine insulin (0.01 mg/ml, Gibco, Thermo). CAOV-3 (#SCSP-570, National Collection of Authenticated Cell Cultures) were maintained in 10% FBS-supplemented DMEM (Gibco, Thermo). SK-OV-3 (#TCHu185, National Collection of Authenticated Cell Cultures) were cultured in McCoy<apos;>s 5A Medium that was supplemented with 10% FBS (Gibco, Thermo). All cell lines aforementioned were cultured at 37°C an incubator containing a humidified atmosphere with 5% CO_2_.

### Cell transfection and treatment

Firstly, to knockdown the expression of ADAMTS9-AS1, si-NC (siRNA negative control), si-ADAMTS9-AS1-1 (siRNA 1 target ADAMTS9-AS-1), si-ADAMTS9-AS1-2 (siRNA 2 target ADAMTS9-AS-1) were purchased from RioBio (Guangzhou, China). The sequences were shown below: si-ADAMTS9-AS1-1 sequences: 5’-CCAUACUGAUACAGCCAAATT-3’; The si-ADAMTS9-AS1-2 sequences: 5’-CCUAACGACAAGGUCCUAUTT-3’; The si-NC sequences:5’-GUGAGCGAGGCAUAGAACGCAU AUG-3’. Then, si-NC, si-ADAMTS9-AS1-1, and si-ADAMTS9-AS1-2 were transfected into OVCAR3 and CAOV-3 cells for 24 h using lipofectamine 2000 (#BL623B, Biosharp). Cells were collected for further quantitative real-time polymerase chain reaction (qRT-PCR).

To investigate ferroptosis mechanism, OVCAR3 and CAOV-3 cells were firstly transfected with si-NC or si-ADAMTS9-AS1-2, with the best interference effect, for 24 h using lipofectamine 2000; then cells were treated with 30 nM ferroptosis inhibitor Fer-1 (#A4371, APExBIO) for 48 h [^25–27^]. The specific groups were as follows: si-NC, si-ADAMTS9-AS1-2, si-ADAMTS9-AS1-2+ Fer-1.

To investigate the role of miR-587 in EOC progression, miR-587 mimics negative control (miR-NC) and miR-587 mimics were transfected with OVCAR3 and CAOV-3 cells for 24 h or 48 h. The specific groups were miR-NC and miR-587 mimics. miR-NC and miR-587 mimics purchased from RioBio.

To verify the potential regulation mechanism of ADAMTS9-AS1, OVCAR3 and CAOV-3 cells were transfected with si-NC+NC inhibitor (miR-587 inhibitor negative control), si-ADAMTS9-AS1-2+ NC inhibitor, si-ADAMTS9-AS1-2+ miR-587 inhibitor using lipofectamine 2000 for 24 h or 48 h. Then cells were collected for further analysis. NC inhibitor and miR-587 inhibitor purchased from RioBio.

### Cell counting kit-8 (CCK-8) assay

As previous study, we used CCK-8 to evaluate the cell activity or proliferation [12]. After transfection for 24 h, OVCAR3 and CAOV-3 cells were solution (resuspended with 0.5% trypsin (Gibco) and plated in 96-well plates (5 × 10^3^ cell/well). Then cultured for indicated time, and added 10 μL CCK8 (G4103, Servicebio, China) into each well for another 2 h. The absorbance values were detected at 450 nm by microplate reader (Infinite M200, Tecan, Austria).

### Clone formation assay

OVCAR3 and CAOV-3 cells with the indicated treatment were placed in 6-well plate (1,000 cells per well). The cells were placed in a 37°C 5% CO_2_ and saturated humidity incubator for 2 to 3 weeks, during which liquid was appropriately changed. Then phosphate-buffered saline solution (PBS) was used to wash the colonies, stained with 0.1% crystal violet for 15 min. The clone formation ability was observed under a microscope (Carl Zeiss, German) and counted using ImageJ software.

### Transwell assay

OVCAR3 and CAOV-3 cells migration ability was examined by Transwell (BD Biosciences) as previous study with a few revision [12]. The upper chambers were filled with 200 μL of serum-free RPMI-1640 medium (for OVCAR3) or DMEM medium (for CAOV-3) containing 5 × 10^4^ cells. And 600 μL of culture medium was added to the lower compartments. After 24 h, we discarded the chamber culture medium and washed them twice with PBS. The cells on the upper ventricle were wiped off with a wet cotton swab. Cells were fixed 4% paraformaldehyde for 10 min and stained with 0.1% crystal violet for 5 min. Stained cells number was counted with a light microscope (magnification, ×200) in 5 random fields of view.

### Detection of Fe^2+,^ iron and reactive oxygen species (ROS) level

Iron assay kit (#MAK025, Sigma) and ROS assay kit (#S0033S, Beyotime) were performed to examine the intracellular Fe^2+^, Iron and ROS levels in OVCAR3 and CAOV-3 cells according to manufacturers’ instructions.

### qRT-PCR

As previous study, we used qRT-PCR to evaluate the gene expression [12]. Trizol reagent (B131905, Biosntech, China) isolated RNAs from cells. Total RNAs were subjected into cDNA reverse transcription (#K1622, Thermo). qRT-PCR assay was performed using SYBR-Green (#P122, Vazyme, China). Using U6 or GAPDH as internal gene, the relative expression was calculated by 2^−ΔΔCt^. The primers were synthesized at Sangon Biotech (Shanghai, China). Primer sequences were as follows: ADAMTS9-AS1-F: 5′-CCATCACTAATCGCCA GGAT-3′, ADAMTS9-AS1-R: 5′-CTGTTGTGGAGTTGCCCTTC-3′; miR-587-F: 5′-CCAGGCAAGAGAGAGTTGCTG-3′, miR-587-RT: 5′-AGTCACAGGTGCAGACACATT-3′; SLC7A11-F: 5′-TGCTGGGCTGATTTTATCTTCG-3′, SLC7A11-R: 5′-GAAAGGGCAACCATGAAGAGG-3′; GAPDH-F: 5′- GAAGGTGAAGGTCGGAGTC-3′, GAPDH-R: 5′- GAAGATGGTGATGGGATTTC-3′; U6-F: 5′-CTCGCTTCGGCAGCACA-3′, U6-R: 5′-AACGCTTCACGAATTTGCGT-3′.

### Western blotting

As previous study, we used western blotting to evaluate the gene protein expression [12]. RIPA lysis buffer (#P0013B, Beyotime) extracted total protein from cells according to the instructions and quantified according to BCA protein assay Kit (BL521A, Biosharp). The protein supernatant was mixed with sodium dodecyl sulfate, sodium salt polyacrylamide gelelectrophoresis loading buffer and then bathed in boiling water for 5 min. Proteins were adsorbed on polyvinylidene fluoride membranes by gel electrophoresis and sealed in 5% skim milk solution for 2 h. Then SLC7A11, GPX4, β-actin and GAPDH (Abcam) antibodies were cultured overnight at 4°C, and secondary antibody was incubated for 2 h at 25°C. Using β-actin or GAPDH as internal reference, protein level was measured by ECL reagent (#WBULS0100, Sigma, USA).

### Subcellular fractionation assay

As previous study, we used subcellular fractionation assay to confirm the location of ADAMTS9-AS1 [12]. The lncLocator (http://www.csbio.sjtu.edu.cn/bioinf/lncLocator/) predicted ADAMTS9-AS1 subcellular distribution. The prediction was verified by Cytoplasmic and Nuclear RNA Purification kit (#21,000, Norgen Biotek). In brief, OVCAR3 and CAOV-3 cells were treated with 200 μL ice-cold cell fractionation buffer for 5 min to separate the cytoplasm and nucleus. And then ADAMTS9-AS1 expression in cytoplasm and nucleus was examined by qRT-PCR.

### Bioinformatics prediction and dual-luciferase reporter gene assay

As previous study, we used dual-luciferase reporter gene assay to verify the binding site between ADAMTS9-AS1 and miR-587, and the binding site between SLC7A11 and miR-587 [12]. miRanda-3.3a software predicted the binding sequences between ADAMTS9-AS1 and miR-587, miR-587 and SLC7A11. Then wild type (WT) and mutant type (MUT) sequences of ADAMTS9-AS1 (wt1: 5’-taGGCACATCCTTTATGGAAt-3’; wt2: 5’-ggaACCCCTTACATGTGGAAt-3’; mut1: 5‘-taCCGAATCGCAAGTATTTGt-3’; mut2: 5‘-ggaCACGCCATGACTGAATTt-3’) and SLC7A11 (wt1: 5’-gtttgTTTTCATCTTATGGAAa-3’; wt2: 5’-gcatgtgcTTTTGTATGGAAt-3’; mut1: 5’-gtttgAGTCATTCGAGAATTTa-3’; mut2: 5’-gcatgtgcAGTAGATATAGGt-3’) were synthesized and inserted into pmirGLO (Promega) by RioBio. OVCAR3 and CAOV-3 cells were placed in a 24-well plate (5 × 10^4^ cells/well) and cotransfected miR-587 mimics (or miR-587 mimics negative control (miR-NC)) and wt (or mut) plasmid of ADAMTS9-AS1 (or SLC7A11. After 48 h transfection, cells were collected for the detection of luciferase activities using luciferase reporter assay kit (#E1910, Promega, USA).

### RNA immunoprecipitation (RIP) assay

As previous study, we used RIP assay to confirm whether Ago2 can enriched ADAMTS9-AS1, miR-587, and SLC7A11 [12]. The EZ-Magna RIP™ kit (#17-701, EMD Millipore) was used in the assay. The cells were lysed in RIP lysis buffer, and treated with magnetic beads coupled to an anti-Ago2 antibody or anti-rabbit IgG at 4°C overnight. Besides, 10 μL whole-cell lysates was used as Input and served as the positive control. IgG was used as negative control. The relative enrichment of ADAMTS9-AS1, miR-587, and SLC7A11 in the immunoprecipitated RNAs was detected via qRT-PCR.

### Statistical analysis

Data was quantified by means ± standard deviation (SD) and compared using Student<apos;>s t-test or one-way analysis of variance (ANOVA) (P < 0.05 as statistically significant) based on SPSS 16.0 software.

## Results

In our study, we speculated that ADAMTS9-AS1 regulates miR-587/SLC7A11 axis to affect cell proliferation and migration by inhibit ferroptosis in EOC progression. To solve this problem, we firstly investigate the function of ADAMTS9-AS1 on cell proliferation, migration, and ferroptosis in EOC. Then we confirmed the regulation relationship of ADAMTS9-AS1, miR-587, and SLC7A11. Finally, we conducted rescue experiments to verify the potential regulation mechanism that ADAMTS9-AS1 regulated miR-587/SLC7A11 axis to affect cell function by inhibiting ferroptosis in EOC progression. Our study was intended to provide new drug targets for EOC diagnosis and treatment.

### 1. Knocking down ADAMTS9-AS1 could inhibit EOC cells proliferation and migration by promoting ferroptosis

**Figure 1.**
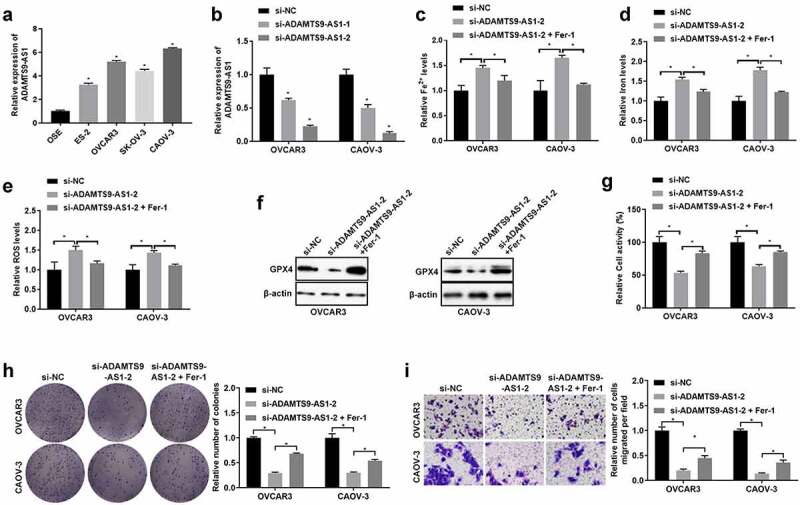
Knocking down long non-coding RNA ADAMTS9-AS1 could inhibit epithelial ovarian cancer cells proliferation and migration by promoting ferroptosis. (A). The long non-coding RNA ADAMTS9-AS1 (ADAMTS9-AS1) expression in different epithelial ovarian cancer cells (including ES-2, OVCAR3, SK-OV-3, and CAOV-3) and human ovarian surface epithelial cell line OSE cells was detected by quantitative real-time polymerase chain reaction (qRT-PCR). N = 3, one-way analysis of variance. (B). Interference effect of ADAMTS9-AS1 in OVCAR3 and CAOV-3 cells after transfection with siRNA negative control (si-NC), siRNA 1 target ADAMTS9-AS1 (si-ADAMTS9-AS1-1), or siRNA 2 target ADAMTS9-AS1 (si-ADAMTS9-AS1-2) for 24 h was measured by qRT-PCR. N = 3, one-way analysis of variance. (C-I) OVCAR3 and CAOV-3 cells were firstly transfected with si-NC or si-ADAMTS9-AS1-2 for 24 h, followed with or without 30 nM ferroptosis inhibitor Fer-1 treatment for another 48 h. Then Iron assay kit was used to determine Fe^2+^ (C) and Iron (D) expressions; ROS assay kit was used examine ROS expression (E); Western blotting detected glutathione peroxidase 4 (GPX4) expression (F); Cell Counting Kit-8 was used to detect cells viability (G); Clone formation assay examined proliferation ability, left is the representative pictures and right is the analysis from three dependent experiment (H) Transwell assay measured migration ability, left is the representative pictures and right is the analysis from three dependent experiment (I). N = 3, one-way analysis of variance. * P < 0.05.

Firstly, OSE and EOC cells (ES-2, OVCAR3, CAOV-3 and SK-OV-3 cells) were cultured and collected, the ADAMTS9-AS1 expression was assessed by qRT-PCR. Results indicated that ADAMTS9-AS1 was highly expressed in EOC cells, and had the highest expression in OVCAR3 and CAOV-3 cells (Figure 1A). Therefore, OVCAR3 and CAOV-3 cells were selected for further study. Then, OVCAR3 and CAOV-3 cells were transfected with siRNAs target ADAMTS9-AS1 for 24 h to silence the ADAMTS9-AS1 expression, si-NC as the control group. The results showed the synthesized si-ADAMTS9-AS1 (including si-ADAMTS9-AS1-1 and si-ADAMTS9-AS1-2) had interference effect, and si-ADAMTS9-AS1-2 with the best interference effect was used in subsequent experiments (Figure 1B). To investigate ferroptosis mechanism, we firstly transfected si-ADAMTS9-AS1-2 (or si-NC) into OVCAR3 and CAOV-3 cells for 24 h, then 30 nM ferroptosis inhibitor Fer-1 was used to treat cells for another 48 h. After detection using iron assay kit, ROS assay kit, and western blotting, we found that compared with si-NC group, Fe^2+^, iron, and ROS increased, GPX4 expression decreased after knocking down ADAMTS9-AS1; Fe^2+^, iron, and ROS decreased, GPX4 expression increased in si-ADAMTS9-AS1-2+ Fer-1 group when compared to silence ADAMTS9-AS1 alone group (Figure 1C-1F). In addition, CCK-8, clone formation assay, and Transwell assay results indicated that compared to si-NC group, knocking down ADAMTS9-AS1 resulted in decreased cell viability and decreased cell proliferation and migration abilities; Fer-1 increased cell viability and increased cell proliferation and migration abilities in si-ADAMTS9-AS1 cells (Figure 1G-1I). These results suggested knocking down ADAMTS9-AS1 could inhibit EOC cells proliferation and migration by promoting ferroptosis.

### 2. ADAMTS9-AS1 negatively regulated miR-587 expression in EOC cells

**Figure 2.**
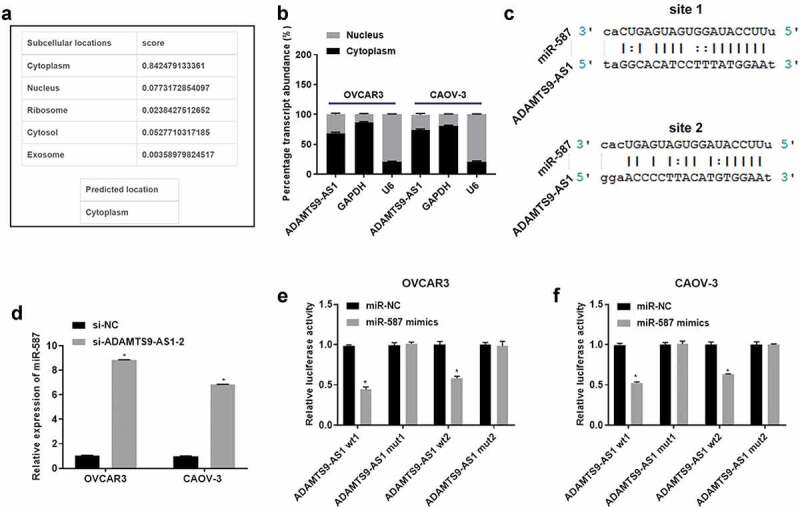
Long non-coding RNA ADAMTS9-AS1 negatively regulated micoRNA-587 expression in epithelial ovarian cancer. (A). LncLocator predicted long non-coding RNA ADAMTS9-AS1 (ADAMTS9-AS1) subcellular distribution. (B). Quantitative real-time polymerase chain reaction (qRT-PCR) measured the expression ratio of ADAMTS9-AS1 in cytoplasm and nucleus of OVCAR3 and CAOV-3 cells after reseparating the cytoplasm and nucleus using Cytoplasmic and Nuclear RNA Purification kit. N = 3 (C). miRanda-3.3a software predicted ADAMTS9-AS1 binding sites to micoRNA-587 (miR-587). (D). qRT-PCR determined miR-587 expression after transfected with siRNAs, si-ADAMTS9-AS1-2 (siRNA targetADAMTS9-AS1) or si-NC (siRNA negative control). N = 3, Student<apos;>s t-test. (E-F). The binding regulatory relationship between ADAMTS9-AS1 and miR-587 was confirmed by dual-luciferase detection in OVCAR3 (E) and CAOV-3 (F) cells after transfection with miR-587 mimics negative control (miR-NC)+ADAMTS9-AS1 wild type vector 1 (ADAMTS9-AS1 wt1), miR-NC+ADAMTS9-AS1 mutant type vector 1 (ADAMTS9-AS1 mut1), miR-NC+ADAMTS9-AS1 wild type vector 2 (ADAMTS9-AS1 wt2), miR-NC+ADAMTS9-AS1 mutant type vector 2 (ADAMTS9-AS1 mut2), miR-587 mimics+ADAMTS9-AS1 wt1, miR-587 mimics+ADAMTS9-AS1 mut1, miR-587 mimics+ADAMTS9-AS1 wt2, and miR-587 mimics+ADAMTS9-AS1 mut2 respectively for 48 h. N = 3, Student<apos;>s t-test. * P < 0.05.

Next, we used lncLocator to predict ADAMTS9-AS1 subcellular distribution. Among cytoplasm, nucleus, ribosome, cytosol, and exosome, the subcellular localization prediction score of ADAMTS9-AS1 was the highest in cytoplasm (Figure 2A). Cytoplasmic and Nuclear RNA Purification kit separated the cytoplasm and nucleus of OVCAR3 and CAOV-3 cells, then the expression of ADAMTS9-AS1, GAPDH, and U6 in cytoplasm and nucleus was detected using qRT-PCR. GAPDH and U6 was used as control of cytoplasm and nucleus separately. Results showed that GAPDH expression in cytoplasm or U6 expression in nucleus possessed above 80% respectively, confirming that cytoplasm and nucleus separation was successfully (Figure 2B). And ADAMTS9-AS1 expression in cytoplasm possessed about 60%, confirming that ADAMTS9-AS1 was mainly located in the cytoplasm of OVCAR3 and CAOV-3 cells (Figure 2B). To investigate the potential mechanism of ADAMTS9-AS1, miRanda-3.3a software was used to predict downstream miRNA. We found that ADAMTS9-AS1 had two binding sites to miR-587 (Figure 2C). miR-587 have been reported exert promote or inhibit role in different tumors [15,16,28]. Therefore, we detected the miR-587 expression in OVCAR3 and CAOV-3 cells after knockdown ADAMTS9-AS1. Results showed that miR-587 expression was promoted after knocking down ADAMTS9-AS1 (Figure 2D). In addition, we performed dual-luciferase detection to confirm the binding site between ADAMTS9-AS1 and miR-587. Results showed that ADAMTS9-AS1 regulated miR-587 expression by binding to site 1 and site 2 (Figure 2E-F). These results showed that ADAMTS9-AS1 negatively regulated miR-587 expression.

### 3. Overexpression of miR-587 targeted regulation of SLC7A11 in EOC cells

**Figure 3.**
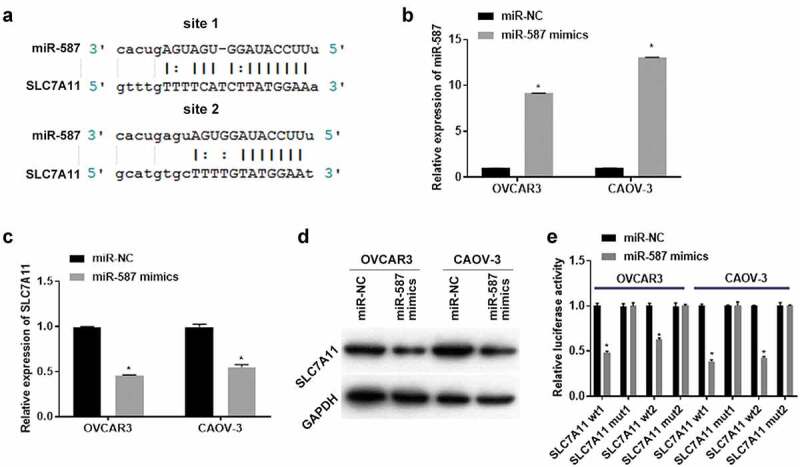
Overexpression of micoRNA-587 targeted regulation of solute carrier family 7 member 11 in epithelial ovarian cancer. (A). miRanda-3.3a software predicted micoRNA-587 (miR-587) binding sites to solute carrier family 7 member 11 (SLC7A11). (B). Quantitative real-time polymerase chain reaction (qRT-PCR) detected the miR-587 expression in OVCAR3 and CAOV-3 cells after transfected with miR-587 mimics negative control (miR-NC) or miR-587 mimics for 24 h. N = 3, Student<apos;>s t-test. (C). qRT-PCR) detected the SLC7A11 expression. OVCAR3 and CAOV-3 cells were transfected with miR-NC or miR-587 mimics for 24 h. N = 3, Student<apos;>s t-test. (D) Western blotting measured SLC7A11 expression. OVCAR3 and CAOV-3 cells were transfected with miR-NC or miR-587 mimics for 48 h. (E). Dual-luciferase detection confirmed the binding regulatory relationship between SLC7A11 and miR-587. OVCAR3 and CAOV-3 cells transfected with miR-587 miR-NC+SLC7A11 wild type vector 1 (SLC7A11 wt1), miR-NC+SLC7A11 mutant type vector 1 (SLC7A11 mut1), miR-NC+SLC7A11 wild type vector 2 (SLC7A11 wt2), miR-NC+SLC7A11 mutant type vector 2 (SLC7A11 mut2), miR-587 mimics+SLC7A11 wt1, miR-587 mimics+SLC7A11 mut1, miR-587 mimics+SLC7A11 wt2, and miR-587 mimics+SLC7A11 mut2 respectively for 48 h. N = 3, Student<apos;>s t-test. * P < 0.05.

To investigate the effect of miR-587 on the downstream mRNA, miRanda-3.3a software prediction indicated that there were two binding sites between SLC7A11 and miR-587 (Figure 3A). Then OVCAR3 and CAOV-3 cells were transfected with miR-NC (or miR-587 mimics) for 24 h (for qRT-PCR detection) or 48 h (for western blotting detection). qRT-PCR results showed that the miR-587 mimics had an overexpression effect, compared to the miR-NC group (Figure 3B). In addition, overexpression of miR-587 inhibited SLC7A11 mRNA and protein levels, compared to the miR-NC group (Figure 3C-3D). These results indicated that miR-587 regulates SLC7A11 expression maybe through the potential binding site. Therefore, we performed dual-luciferase detection to confirm the binding site between SLC7A11 and miR-587. Results showed that miR-587 regulates SLC7A11 expression by binding to site 1 and site 2 (Figure 3E). These indicated overexpression of miR-587 targeted regulation of SLC7A11.

### 4. Overexpression of miR-587 promoted ferroptosis and inhibited EOC cells proliferation and migration

**Figure 4.**
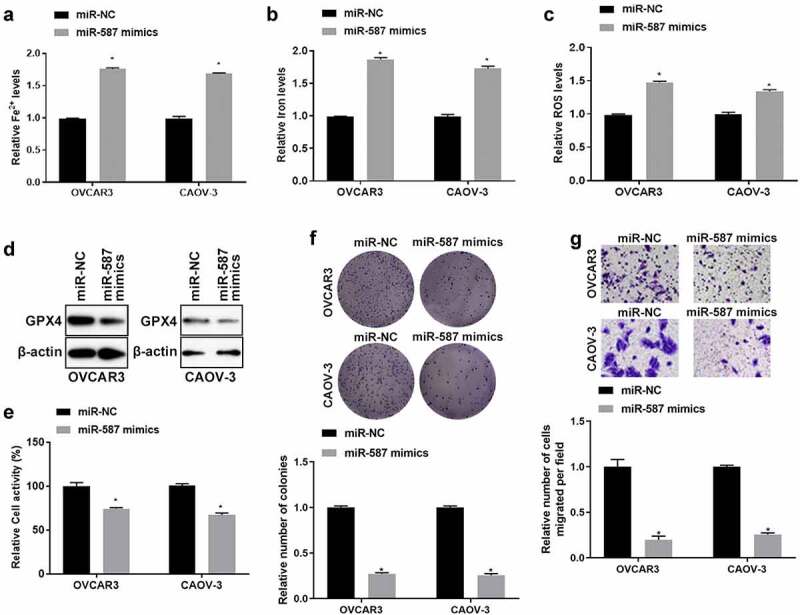
Overexpression of micoRNA-587 promoted ferroptosis and inhibited epithelial ovarian cancer cells proliferation and migration. Iron assay kit measured Fe^2+^ (A) and Iron (B) expression. OVCAR3 and CAOV-3 cells were transfected with miroRNA-587 (miR-587) mimics negative control (miR-NC) or miR-587 mimics for 24 h. N = 3, Student<apos;>s t-test. (C). ROS assay kit measured ROS expression. OVCAR3 and CAOV-3 cells were transfected with miR-NC or miR-587 mimics for 24 h. N = 3, Student<apos;>s t-test. (D). Western blotting detected glutathione peroxidase 4 (GPX4) expression. OVCAR3 and CAOV-3 cells were transfected with miR-NC or miR-587 mimics for 48 h. (E). Cell Counting Kit-8 detected cell activity. OVCAR3 and CAOV-3 cells were transfected with miR-NC or miR-587 mimics for 24 h. N = 3, Student<apos;>s t-test. (F). Clone formation assay tested proliferation ability, left is the representative pictures and right is the analysis from three dependent experiment. OVCAR3 and CAOV-3 cells were transfected with miR-NC or miR-587 mimics for 24 h. N = 3, Student<apos;>s t-test. F. Transwell assay detected migration ability, left is the representative pictures and right is the analysis from three dependent experiment. OVCAR3 and CAOV-3 cells were transfected with miR-NC or miR-587 mimics for 24 h. N = 3, Student<apos;>s t-test. * P < 0.05.

Next, we further investigated miR-587 effects on ferroptosis and cell function in EOC. OVCAR3 and CAOV-3 cells were transfected with miR-NC or miR-587 mimics for 24 h (for the detection of Fe^2+^ levels, Iron expression, ROS levels, cell activity, cell proliferation ability, and cell migration ability) or 48 h (for western blotting assay). Figure 4A and 4B showed that Fe^2+^ and Iron expressions increased in OVCAR3 and CAOV-3 cells after miR-587 overexpression using Iron assay kit. Besides, ROS assay kit detected results also indicated that ROS levels increased after miR-587 overexpression (Figure 4C). Moreover, western blotting results showed that GPX4 expression was decreased after miR-587 overexpression (Figure 4D). The above results indicated that overexpression of miR-587 promoted ferroptosis. Then we investigated the cell function changes. CCK-8 result showed the cell viability was down-regulated in miR-587 overexpressed OVCAR3 and CAOV-3 cells, compared with their respective miR-NC group (Figure 4E). Additionally, cells proliferation and migration abilities were weakened after miR-587 was overexpression (Figure 4F-4G). These results revealed that overexpression of miR-587 promoted ferroptosis and inhibited EOC cells proliferation and migration.

### 5. ADAMTS9-AS1 regulated SLC7A11 expression through miR-587 in EOC cells

**Figure 5.**
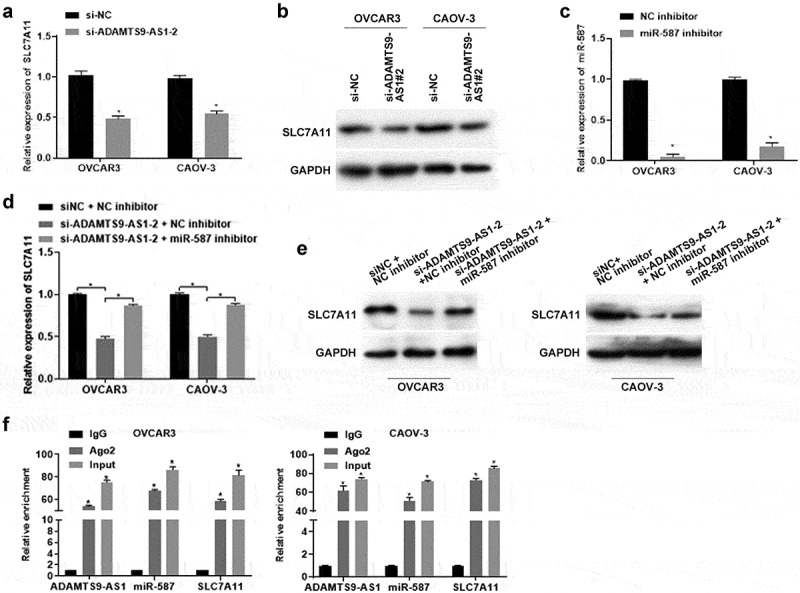
Long non-coding RNA ADAMTS9-AS1 regulated solute carrier family 7 member 11 expression through micoRNA-587 in epithelial ovarian cancer. (A). Quantitative real-time polymerase chain reaction (qRT-PCR) measured solute carrier family 7 member 11 (SLC7A11) expression. OVCAR3 and CAOV-3 cells were transfected with siRNA negative control (si-NC) or siRNA 2 target long non-coding RNA ADAMTS9-AS-1 (si-ADAMTS9-AS1-2) for 24 h. N = 3, Student<apos;>s t-test. (B). Western blotting detected SLC7A11 expression. OVCAR3 and CAOV-3 cells were transfected with si-NC or si-ADAMTS9-AS1-2 for 48 h. (C). qRT-PCR detected the micoRNA-587 (miR-587) expression. OVCAR3 and CAOV-3 cells were transfected with miR-587 inhibitor negative control (NC inhibitor) or miR-587 inhibitor for 24 h. N = 3, Student<apos;>s t-test. (D) qRT-PCR detected SLC7A11 expression. OVCAR3 and CAOV-3 cells were transfected with si-NC+NC inhibitor, si-ADAMTS9-AS1-2+ NC inhibitor, si-ADAMTS9-AS1-2+ miR-587 inhibitor for 24 h. N = 3, Student<apos;>s t-test. (E). Western blotting determined SLC7A11 expression. OVCAR3 and CAOV-3 cells were transfected with si-NC+NC inhibitor, si-ADAMTS9-AS1-2+ NC inhibitor, si-ADAMTS9-AS1-2+ miR-587 inhibitor for 48 h. (F). qRT-PCR detected the expression of ADAMTS9-AS1, miR-587, and SLC7A11. OVCAR3 and CAOV-3 cells were collected for RNA immunoprecipitation using anti-Ago2 antibody or anti-rabbit IgG. N = 3, one-way analysis of variance. * P < 0.05.

To explore whether ADAMTS9-AS1 regulates SLC7A11 expression through miR-587, we firstly detected SLC7A11 expression in ADAMTS9-AS1 knockdown EOC cells. OVCAR3 and CAOV-3 cells were transfected with si-NC or si-ADAMTS9-AS1-2 for 24 h (for qRT-PCR detection) or 48 h (for western blotting). SLC7A11 mRNA and protein levels decreased after knocking down ADAMTS9-AS1 (Figure 5A and 5B). Afterwards, we conducted rescue experiment to test SLC7A11 expression. OVCAR3 and CAOV-3 cells were transfected with si-NC+NC inhibitor, si-ADAMTS9-AS1-2+ NC inhibitor, si-ADAMTS9-AS1-2+ miR-587 inhibitor for 24 h (for qRT-PCR) or 48 h (for western blotting assay), Results showed that SLC7A11 mRNA and protein expression decreased in si-ADAMTS9-AS1-2+ NC inhibitor group compared with si-NC+NC inhibitor group; while adding miR-587 inhibitor simultaneously could increase SLC7A11 mRNA and protein level in ADAMTS9-AS1 interference cells (Figure 5D-5E). The above results indicated that ADAMTS9-AS1 regulated SLC7A11 expression through miR-587. Finally, we performed RIP experiment in OVCAR3 and CAOV-3 cells followed with qRT-PCR detection the expression of ADAMTS9-AS1, miR-587, and SLC7A11. Relative to IgG group, the expression of ADAMTS9-AS1, miR-587, and SLC7A11 was increased in Ago2 (or Input) group (Figure 5F). This result indicated that Ago2 enriched ADAMTS9-AS1, miR-587, and SLC7A11. In another word, miR-587 was as a sponge for ADAMTS9-AS1 and SLC7A11 base on the results of Figure 2(Figure 3andFigure 5. These results further confirmed that ADAMTS9-AS1 regulated SLC7A11 expression through miR-587.

### 6. ADAMTS9-AS1 affected ferroptosis, proliferation and migration of EOC cells through miR-587

**Figure 6.**
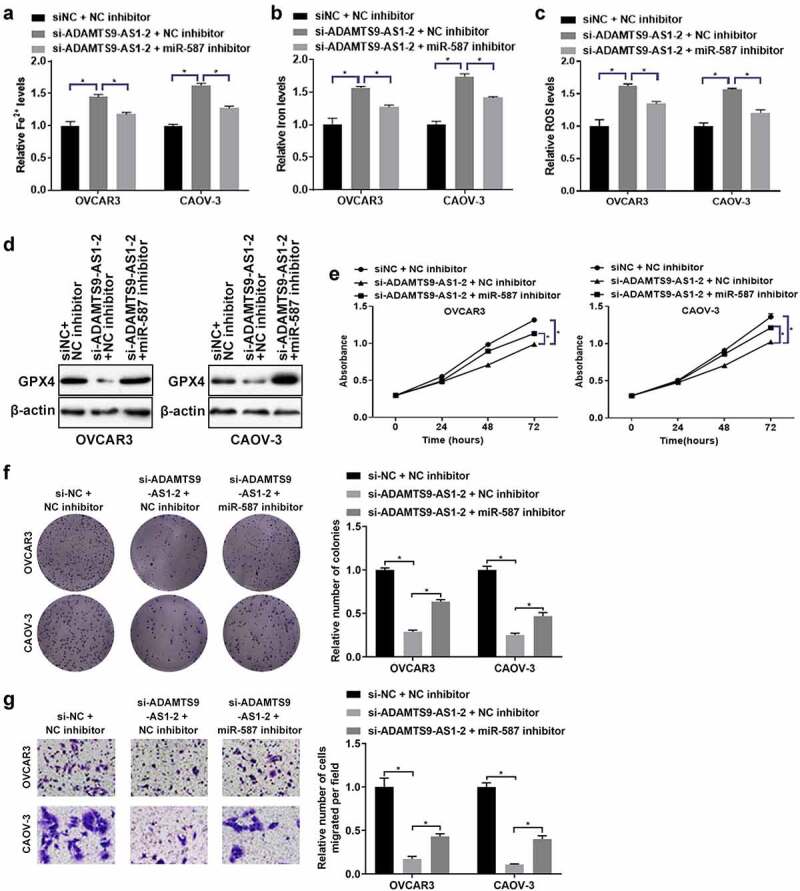
Long non-coding RNA ADAMTS9-AS1 affected ferroptosis, proliferation and migration of epithelial ovarian cancer cells through microRNA-587. OVCAR3 and CAOV-3 cells were transfected with siRNA negative control (si-NC)+micoRNA-587 inhibitor negative control (NC inhibitor), siRNA 2 target long non-coding RNA ADAMTS9-AS-1 (si-ADAMTS9-AS1-2)+NC inhibitor, si-ADAMTS9-AS1-2+ micoRNA-587 (miR-587) for 24 h (for the detection of Fe^2+^ levels, Iron expression, ROS levels, cell activity, cell proliferation ability, and cell migration ability) or 48 h (for western blotting). Iron assay kit measured Fe^2+^ (A) and Iron (B) expressions. N = 3, one-way analysis of variance. (C). ROS assay kit determined ROS expression. N = 3, one-way analysis of variance. (D). Western blotting tested glutathione peroxidase 4 (GPX4) expression. (E). Cell Counting Kit-8 detected cell proliferation at 24, 48, and 72 h. N = 3, one-way analysis of variance. (F). Clone formation assay detected proliferation ability, left is the representative pictures and right is the analysis from three dependent experiment. N = 3, one-way analysis of variance. (G). Transwell assay tested migration ability, left is the representative pictures and right is the analysis from three dependent experiment. N = 3, one-way analysis of variance. * P < 0.05.

Finally, we further investigated whether ADAMTS9-AS1 affected ferroptosis and cell function in EOC through miR-587. OVCAR3 and CAOV-3 cells were transfected with si-NC+NC inhibitor, si-ADAMTS9-AS1-2+ NC inhibitor, and si-ADAMTS9-AS1-2+ miR-587 inhibitor for 24 h (for the detection of Fe^2+^ levels, Iron expression, ROS levels, cell activity, cell proliferation ability, and cell migration ability) or 48 h (for western blotting). Relative to si-NC+NC inhibitor group, knocking down ADAMTS9-AS1 increased the expression of Fe^2+^ and Iron; adding miR-587 inhibitor at the same time reduced the increase of Fe^2+^ and Iron induced by knocking down ADAMTS9-AS1 (Figure 6A-6B). ROS results showed that the ROS increased after ADAMTS9-AS1 was interfered when compared with si-NC+NC inhibitor group; the increase of ROS caused by si-ADAMTS9-AS1-2 was decreased by interfering miR-587 expression simultaneously (Figure 6C). In addition, western blotting results indicated that the GPX4 expression was decreased after silence of ADAMTS9-AS1 when compared with si-NC+NC inhibitor group; adding miR-587 inhibitor at the same time increased GPX4 expression in ADAMTS9-AS1 knockdown cells (Figure 6D). Cell function experiments showed that the abilities of cell proliferation (72 h), clone formation, and migration were decreased after knocking down ADAMTS9-AS1; transfected with miR-587 inhibitor at the same time increased the abilities of cell proliferation (72 h), clone formation, and migration in ADAMTS9-AS1 knock down cells (Figure 6E-6G). All in all, silence of ADAMTS9-AS1 promoted ferroptosis and inhibited the abilities of proliferation and migration in EOC cells; however, these effects were reversed by miR-587 inhibitor, suggesting that ADAMTS9-AS1 affected ferroptosis, proliferation and migration of EOC cells through miR-587.

## Discussion

With the further development of the treatment of EOC to personalized medicine, new methods for early diagnosis and prevention using molecular genomics are under development [29]. In this study, we investigated whether ADAMTS9-AS1 could regulate SLC7A11 expression and inhibit ferroptosis by sponging miR-587 in EOC progression through in vitro cell experiments. Our results indicated that ADAMTS9-AS1 attenuated ferroptosis by targeting miR-587/SLC7A11 axis in EOC. At present, there is no reported study on the ADAMTS9-AS1/miR-587/SLC7A11 axis role in regulating ferroptosis of EOC cells, which is our innovation.

lncRNA is considered to be a regulator of gene expression, and lncRNA disorder is involved in many cancers progression, including EOC [30]. It has been reported that ADAMTS9-AS1 overexpression preferentially affected genes related to proliferation and migration [31]. In hepatocellular carcinoma, ADAMTS9-AS1 triggered PI3K/AKT/mTOR pathway of liver cancer cells, exacerbated cell proliferation and migration [32]. In non-small cell lung cancer, ADAMTS9-AS1 knockdown inhibited cell proliferation and epithelial-mesenchymal transformation [33]. Wang H, et al found ADAMTS9-AS1 expression was elevated in EOC [10]. Our study found ADAMTS9-AS1 was highly elevated in EOC cells, and the highest expression was found in OVCAR3 and CAOV-3 cells. This was consistent with the research results of Wang H, et al. In addition, our results revealed knocking down ADAMTS9-AS1 inhibited EOC cells proliferation and migration by promoting ferroptosis. This was consistent with findings in other cancers that ADAMTS9-AS1 affected cancer cells proliferation and migration.

ceRNA is a post-transcriptional transcript that is mutually regulated by competing for shared miRNAs [34]. Interaction between ceRNAs through shared miRNAs represents a new gene regulatory layer, which plays an essential role in physiology and cancer and other diseases development [35]. Fang S, et al reported ADAMTS9-AS1 inhibited invasive phenotype of breast cancer cells by sponging miR-513a-5p and regulating ZFP36 ring finger protein [36]. Zhou Z, et al found ADAMTS9-AS1 inhibited prostate cancer progression via regulating miR-142-5p/cyclin D1 [37]. We found ADAMTS9-AS1 negatively regulated miR-587 expression, and overexpression of miR-587 targeted regulation of SLC7A11. In addition, through miRanda-3.3a prediction and dual-luciferase validation, and RIP assay, we confirmed the binding between ADAMTS9-AS1 and miR-587, as well as the binding between miR-587 and SLC7A11, suggesting that ADAMTS9-AS1 regulated SLC7A11 expression through sponging miR-587. Our results revealed ADAMTS9-AS1/miR-587/SLC7A11 played a vital role in EOC through the ceRNA mechanism.

SLC7A11 is an important oncoprotein, which not only plays a role in defending against oxidative stress and ferroptosis, but also plays a role in influencing malignant tumor behavior and tumor microenvironment [38]. Ferroptosis is a new type of programmed non-apoptotic cell death caused by iron-dependent lipid peroxidation after the inactivation of SLC7A11 andsolute carrier family 3 member 2, which is involved in all kinds of diseases [39]. Ferroptosis, as a promising new antitumor strategy, has shown a vital role in EOC [40]. It has been reported in BRCA-proficient OC, inhibition of SLC7A11 resulted in decreased glutathione biosynthesis and promoted lipid peroxidation and ferroptosis [41]. We verified that overexpression of miR-587 targeted regulation of SLC7A11 promoted ferroptosis and inhibited EOC cells proliferation and migration. In addition, ADAMTS9-AS1 regulated SLC7A11 expression through miR-587, thereby affecting ferroptosis, proliferation and migration of EOC cells. This research provides a possibility for EOC treatment in ferroptosis.

However, we still need to further investigate these effects in animal models. Moreover, samples from EOC patient also need collected to verify our conclusion.

## Conclusion

Our study indicated that lncRNA ADAMTS9-AS1 attenuated ferroptosis by targeting miR-587/SLC7A11 axis in EOC. Our study provides a new target for EOC treatment. It also provides a reference for understanding the molecular mechanism of ferroptosis in EOC.
